# Epidemiology, Risk Factors, and Clinical Outcomes of AKI in Pediatric Hematopoietic Stem Cell Transplant Patients

**DOI:** 10.34067/KID.0000000000000410

**Published:** 2024-03-07

**Authors:** Omer S. Ashruf, Zaid Ashruf, Zara Orozco, Matt Zinter, Rolla Abu-Arja, Keval Yerigeri, Imad U. Haq, David C. Kaelber, John Bissler, Rupesh Raina

**Affiliations:** 1 Department of Internal Medicine, Northeast Ohio Medical University, Rootstown, Ohio; 2 Department of Nephrology, Akron Nephrology Associates/Cleveland Clinic Akron General Medical Center, Akron, Ohio; 3 Division of Critical Care Medicine, Department of Pediatrics, School of Medicine, University of California, San Francisco, California; 4 Division of Hematology, Oncology, Blood and Bone Marrow Transplant, Nationwide Children's Hospital, Columbus, Ohio; 5 Department of Pediatrics, The Ohio State University College of Medicine, Columbus, Ohio; 6 Department of Internal Medicine-Pediatrics, The MetroHealth System, Cleveland, Ohio; 7 Center for Clinical Informatics Research and Education, The MetroHealth System and the Departments of Internal Medicine, Pediatrics, and Population and Quantitative Health Sciences, Case Western Reserve University, Cleveland, Ohio; 8 Department of Pediatrics, University of Tennessee Health Science Center and Le Bonheur Children's Hospital, Memphis, Tennessee; 9 Department of Nephrology, Akron Children's Hospital, Akron, Ohio

**Keywords:** AKI, clinical epidemiology, dialysis, ICD-9-CM, pediatric intensive care medicine, pediatric kidney transplantation, pediatric nephrology, pediatrics

## Abstract

**Key Points:**

- The cumulative incidence of AKI diagnosis post–hematopoietic stem cell transplantation was 12.9%. Calcineurin inhibitor use was associated with the highest cumulative incidence, 21.6%, after hematopoietic stem cell transplantation.
- Patients with AKI with hypertension/hypertensive disease had a 30-day survival probability of 63.9% (hazard ratio, 4.86, 95% confidence interval, 3.58 to 6.60).
- Patients with AKI were 2.5 times more likely to experience composite hospitalization and/or mortality at 30 days. Of patients who developed AKI, dialysis dependence has nearly tripled since 2014.

**Background:**

AKI is a common complication in pediatric patients undergoing hematopoietic stem cell transplantation (HSCT), with a reported prevalence ranging from 68% to 84%. Few multicenter pediatric studies comprehensively assess the epidemiologic associations and clinical outcomes associated with AKI development.

**Methods:**

An observational, retrospective analysis was conducted using an aggregated electronic health record data platform. The study population consisted of pediatric patients (age <18 years) who underwent HSCT over a 20-year period. The study groups consisted of patients with an encounter diagnosis of AKI (*n*=713) and those without AKI (*n*=4455). Both groups were propensity matched for age, sex, race, prior cancer diagnosis, and other comorbidities. End points were incidence, mortality risk, clinical outcomes, and prevalence of dialysis dependence. Competing risks analysis, Cox proportional hazard analyses, Kaplan–Meier survival curves, and incidence/prevalence rates were calculated.

**Results:**

After matching, 688 patients were identified. Cumulative incidence of AKI diagnosis post-HSCT was 13.7%. Hypertensive disease, calcineurin inhibitors, and vancomycin were the most prevalent risk factors for AKI, with calcineurin inhibitors showing the highest cumulative incidence (21.6%). Patients with AKI with hypertensive disease had a survival probability of 63.9% at 30 days, followed by calcineurin inhibitors (64.4%) and vancomycin (65.9%). Patients with AKI were 1.7 times more likely to experience composite hospitalization and/or mortality at 30 days. At 365 days post-HSCT, patients with AKI had higher rates of all-cause emergency department visits, intensive care unit admissions, and mechanical ventilation compared with non-AKI. Of patients who developed AKI, the prevalence of dialysis dependence has nearly tripled since 2014.

**Conclusions:**

The findings highlight a strong association between specific risk factors, such as hypertension, calcineurin inhibitor use, and vancomycin use, with increased mortality and adverse clinical outcomes in patients with AKI after HSCT. These results emphasize the need for preventative actions such as 24-hour BP monitoring and discontinuation of potential nephrotoxic medications.

## Visual Abstract

**Figure:**
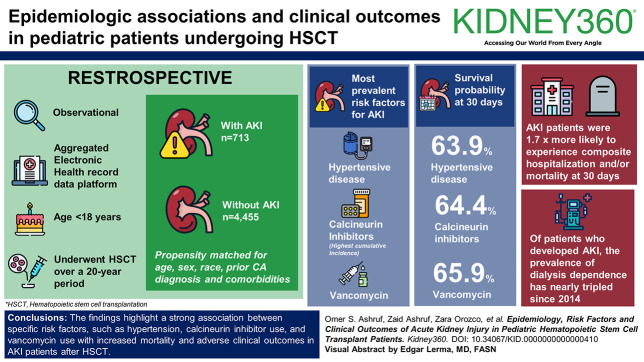

## Introduction

AKI is a complex condition with a variety of molecular and clinical implications, defined generally as sudden decline in kidney function measured by increased serum creatinine levels or decreased urine output within a 3-month period.^1^ The global incidence of AKI among pediatric patients ranges from 26.9% to 41.3% and is a common cause of mortality in this demographic.^1^ In-hospital mortality of pediatric patients with AKI is greater than those without AKI, regardless of stage or severity.^2^ Furthermore, pediatric patients hospitalized with AKI are at increased risk of other unfavorable outcomes including the need for ventilation and RRT, length-of-stay, and long-term development of CKD.^3,4^

Hematopoietic stem cell transplantation (HSCT) is widely applied as a treatment for malignant and nonmalignant conditions, such as hemoglobinopathies, bone marrow failure, and immune deficiencies.^5^ AKI is one of the most common complications in children receiving HSCT, with prevalence as high as 68%–84% in two large-scale studies of pediatric HSCT recipients.^6,7^ A variety of risk factors are described that worsen outcomes in allogeneic HSCT patients with AKI including immune-mediated conditions, such as graft-versus-host disease (GVHD),^8^ hepatic veno-occlusive disease (VOD),^9^ and transplant-associated thrombotic microangiopathy (TMA).^10^ Immunomodulatory agents, calcineurin inhibitors, and/or mammalian target of rapamycin inhibitors are prescribed to prevent GVHD but may beget kidney dysfunction. AKI in patients with GVHD is associated with poor outcomes, including progression to CKD and RRT requirements.^11,12^

There are limited data quantifying epidemiologic characteristics and clinical outcomes in this vulnerable population. In addition, the comprehensive effect of HSCT-induced AKI in pediatric patients and their subsequent dependence on hemodialysis has not been studied on a large scale. By querying aggregated electronic health record (EHR) patient data, we assessed competing risk factors, clinical outcomes, and epidemiology of dialysis dependence in pediatric HSCT patients with AKI.

## Methods

### Database Description

An observational, retrospective analysis was conducted using TriNetX, a platform that aggregates EHR data including patient encounter diagnoses, procedures, medications, laboratory values, and genomic information. The TriNetX Global Collaborative Network consists of approximately 138 million patients across 109 health care organizations. Study participants were selected using relevant International Classification of Diseases, Tenth Revision, Clinical Modification (ICD-10-CM) diagnosis codes, Logical Observation Identifier Names and Codes laboratory codes, current procedural terminology procedure codes, and medical prescription normalized codes.

Per the Case Western Reserve University/MetroHealth System Institutional Review Board, studies that use TriNetX as described herein are exempt from review because the patient data are deidentified and therefore not Human Subject Research. Using the TriNetX platform as such meets the Health Insurance Portability and Accountability Act criteria of deidentified data on the basis of independent expert attestation obtained by the TriNetX corporation. The TriNetX platform has been used extensively in both adult and pediatric patient populations to report epidemiological outcomes and risk assessments in an array of pathologic settings.^13–15^ This study followed the Strengthening the Reporting of Observational Studies in Epidemiology reporting guidelines.^16^ Description of the database is further laid out in the Supplemental Methods.

### Study Design

This study has four key objectives.

1. To analyze the association between HSCT-induced AKI and the following *a priori* identified consensus risk factors: hypertensive disease, hepatic VOD, GVHD, TMA, sepsis, and medications (vancomycin, aminoglycosides, calcineurin inhibitors, and intravenous IgG [IVIG]).^12^
2. To assess the mortality risk associated with aforementioned risk factors.
3. To assess clinical outcomes in patients with AKI compared with control by quantifying rates of hospitalization, emergency department (ED) visitation, intensive care unit (ICU) admission, mechanical intubation/ventilation, and all-cause mortality at 30, 90, 180, and 365 days of HSCT procedure.
4. To determine the incidence and prevalence of dialysis over a 10-year span in patients with AKI.

### Participant Selection and Statistical Analysis

**Figure 1:**
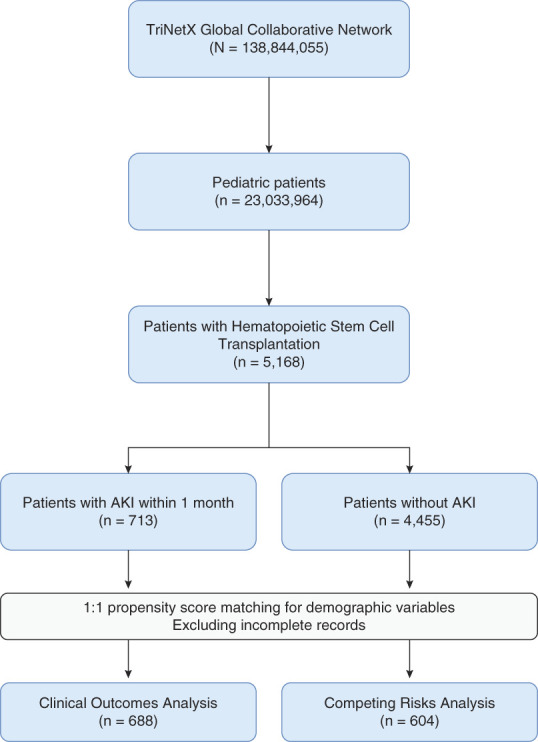
**Flow diagram of study cohorts and inclusion criteria.** Cohorts were 1:1 propensity score matched for age, sex, and race, using nearest neighbor greedy matching algorithm employed by TriNetX Analytics Platform.

The study population consisted of any pediatric patient in the TriNetX database from January 2001 to June 2023 with at least one ICD-10-CM encounter diagnosis for HSCT status (Z94.81), autologous transplant (38,240), or allogenic transplant (38,241). An experimental subgroup was created with patients who had an ICD-10-CM encounter diagnosis of AKI (N17, N17.8, N17.9) within 1 month of HSCT. Those without an encounter diagnosis of AKI within 1 month of HSCT were placed in the control subgroup. A flow diagram of study cohorts is shown in Figure 1.

We used the TriNetX platform's built-in 1:1 propensity score matching function to match patients in the experimental cohort with the closest eligible control counterpart by a nearest neighbor greedy matching algorithm.^17^ Both cohorts were propensity matched for demographic variables such as age, sex, race, and clinical variables including prior cancer diagnosis and CKD/ESKD. All statistical analyses were performed within the TriNetX Analytics Platform. Competing risks analysis, Cox proportional hazard analyses, Kaplan–Meier survival curves, and incidence/prevalence rates were calculated. Information on statistical analysis is provided in the Supplemental Material.

## Results

### Patient Characteristics

This study consisted of 713 eligible pediatric patients who developed AKI within 1 month of HSCT (57.5% male; 53.9% White; average age 6.5±4.7 years; average body mass index 18.9±4.9) and 4455 eligible pediatric patients who underwent HSCT and did not develop AKI (57.7% male; 53.0% White; average age 5.3±4.2 years; average body mass index 17.7±4.1). After propensity matching, 604 patients were included from both cohorts to examine competing risks and 688 patients included to assess clinical outcomes and impact on survival. The cumulative incidence of AKI in the first 30 days was 13.8% (713/5168). Covariates and density function are shown in the Supplemental Figure 1 and Supplemental Table 1. Baseline characteristics are listed in Table 1.

**Table 1:** Baseline characteristics of AKI and control cohorts, before and after matching

Covariates	Before Matching			After Matching		
	AKI (*n*=713)	Control (*n*=4455)	Std Diff.	AKI (*n*=688)	Control (*n*=688)	Std Diff.
**Demographics**						
Age, yr	6.52±4.68	5.34±4.16	0.28	6.45±4.7	6.42±4.71	<0.01
Male, No. (%)	57.50 (410)	57.69 (2570)	<0.01	57.41 (395)	57.85 (398)	<0.01
Female, No. (%)	42.36 (302)	42.00 (1871)	<0.01	42.44 (292)	42.15 (290)	<0.01
White, No. (%)	53.86 (384)	53.04 (2363)	0.02	54.94 (378)	55.23 (380)	<0.01
Black, No. (%)	13.46 (96)	14.25 (635)	0.02	13.66 (94)	12.79 (88)	0.03
Asian, No. (%)	6.45 (46)	4.92 (219)	0.07	5.96 (41)	7.85 (54)	0.07
Other race, No. (%)	13.18 (94)	9.90 (441)	0.10	11.92 (82)	11.63 (80)	<0.01
Hypertension, No. (%)	50.77 (362)	16.41 (731)	0.78	48.98 (337)	50.15 (345)	0.02
Diabetes mellitus, No. (%)	4.91 (35)	1.69 (75)	0.18	4.07 (28)	4.07 (28)	<0.01
CKD, No. (%)	9.12 (65)	1.46 (65)	0.35	7.27 (50)	6.83 (47)	0.02
Proteinuria, No. (%)	9.40 (67)	1.12 (50)	0.38	6.54 (45)	5.09 (35)	0.06
Cancer history, No. (%)	67.46 (481)	52.41 (2335)	0.31	67.0 (461)	68.50 (471)	0.03

1:1 propensity score matching was conducted using a greedy nearest-neighbor matching algorithm with an arbitrary tolerance level set at 0.1. Std Diff., standard difference.

### Cumulative Incidence of AKI Risk Factors

**Figure 2:**
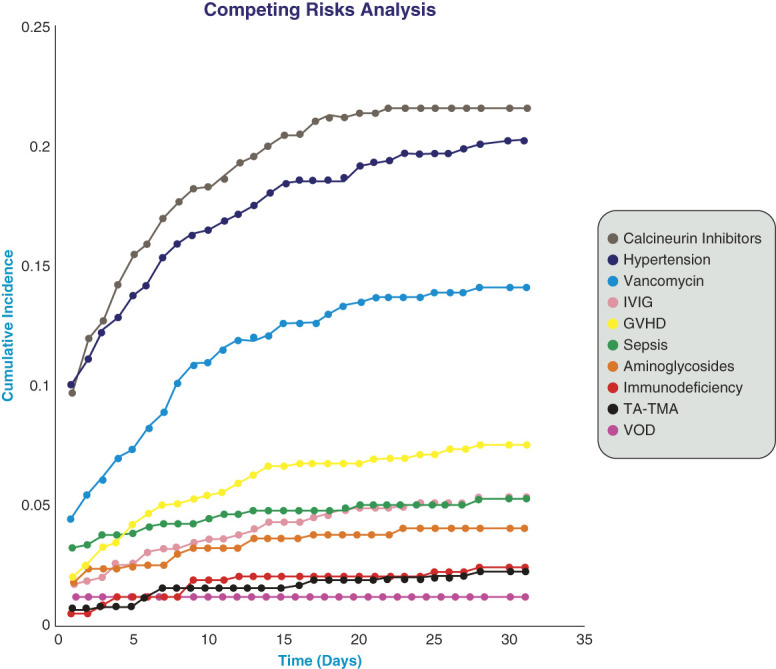
**Aalen–Johansen curves of cumulative incidence values of transplant-related risk factors over 30 days.** The plot of Aalen–Johansen cumulative incidence curves showcases the mutually exclusive competing risks for AKI precipitation, 30 days post-HSCT. Calcineurin inhibitors (21.55%), hypertension/hypertensive disease (20.19%), vancomycin (14.04%), IVIG (7.46%), GVHD (5.21%), sepsis (5.16%), aminoglycosides (3.98%), immunodeficiency because of drugs and external causes (2.44%), TA-TMA (2.26%), and VOD (1.19%). GVHD, graft-versus-host disease; HSCT, hematopoietic stem cell transplantation; IVIG, intravenous IgG; TA-TMA, transplant-associated thrombotic microangiopathy; VOD, veno-occlusive disease.

The cumulative incidences for *a priori* selected AKI risk factors were calculated at the end of the 1-month time window. The following factors are ordered by highest cumulative incidence: calcineurin inhibitors (21.55%), hypertension (20.19%), vancomycin (14.04%), IVIG (7.46%), acute GVHD (5.21%), sepsis (5.16%), aminoglycosides (3.98%), immunodeficiency because of drugs and external causes (2.44%), TMA (2.26%), and hepatic VOD (1.19%), Figure 2.

### 30-Day Survival Stratified by AKI Risk Factors

Within 1 month of HSCT, the following survival probabilities were calculated. Patients with AKI with hypertension/hypertensive disease incurred the greatest mortality risk post-HSCT: 214 of 604 (35.4%) patients with AKI compared with 51 of 604 (8.4%) in control (hazard ratio [HR], 4.86; 3.58 to 6.60), and the survival probability was 63.9%, 27.4% lower than control; 123 of 604 patients with AKI (20.4%) had GVHD compared with 19 of 604 (3.2%) in control (HR, 7.05; 4.35 to 11.44), and the survival probability was 79.1%, 17.6% lower than control; 79 of 604 patients with AKI (13.1%) had sepsis compared with 18 of 604 (3.0%) in control (HR, 4.57; 2.47 to 7.63), and the survival probability was 86.8%, 10.1% lower than control; 57 of 604 patients with AKI (9.4%) had immunodeficiency because of drugs and external causes compared with 26 of 604 (4.3%) in control (HR, 2.22; 1.40 to 3.54), and the survival probability was 90.3%, 5.2% lower than control; 30 of 604 patients with AKI (5.0%) had TMA compared with 10 of 604 (1.7%) in control (HR, 5.07; 2.11 to 12.19), and the survival probability was 94.9%, 4.1% lower than control; 26 of 604 patients with AKI (4.3%) had hepatic VOD compared with 10 of 604 (1.7%) in control (HR, 4.37; 1.80 to 10.61), and the survival probability was 95.6%, 3.3% lower than control.

Of medications prescribed, calcineurin inhibitors (tacrolimus, cyclosporine, voclosporin) were associated with the greatest mortality risk post-HSCT; 211 of 604 patients with AKI (34.9%) were prescribed calcineurin inhibitors compared with 96 of 604 (15.9%) in control (HR, 2.56; 2.01 to 3.26), and the survival probability was 64.4% at 1-month follow-up, 19.1% lower than control; 202 of 604 patients with AKI (33.4%) were prescribed vancomycin compared with 152 of 604 (25.2%) in control (HR, 1.41; 1.14 to 1.74), and the survival probability was 65.9%, 8.0% lower than control; 147 of 604 patients with AKI (24.3%) were administered IVIG compared with 75 of 604 (12.4%) in control (HR, 2.08; 1.57 to 2.74), and the survival probability was 75.0%, 12.1% lower than control; 69 of 604 patients with AKI (11.4%) were prescribed aminoglycosides compared with 29 of 604 (4.8%) in the control cohort (HR, 2.44; 1.58 to 3.76), and the survival probability was 88.3%, 6.7% lower than control; 38 of 604 patients with AKI (6.3%) were prescribed foscarnet compared with 13 of 604 (2.2%) in control (HR, 2.96; 1.58 to 5.56), and the survival probability was 93.6%, 4.2% lower than control; 28 of 604 patients with AKI (4.6%) were prescribed etoposide compared with 13 of 604 (2.2%) in control (HR, 2.16; 1.12 to 4.17), and the survival probability was 95.3%, 2.5% lower than control; 49 of 604 patients with AKI (8.1%) were prescribed methotrexate compared with 54 of 604 (8.9%) in control (HR, 0.89; 0.61 to 1.31), and the survival probability was 91.7%, 1.0% higher than control, Supplemental Table 2.

After HSCT, mean total bilirubin was 1.48 mg/dl in patients with AKI and 0.50 mg/dl in controls (*P* < 0.001). The mean creatinine was 0.57 mg/dl in patients with AKI and 0.32 mg/dl in controls (*P* < 0.001). The mean BUN was 21.46 mg/dl in patients with AKI and 13.81 mg/dl in controls (*P* < 0.001).

### Clinical Outcomes of Patients with AKI

Mortality rate for patients who developed AKI at 30 days was 25 of 688 (3.6%, *P* < 0.001) and patients with AKI were 1.7 times more likely to experience all-cause hospitalization and/or mortality than controls (HR, 1.68; 1.45 to 1.95). All current procedural terminology codes used are listed in Supplemental Tables 3 and 4.

#### At 90-Day Follow-Up

In the non-AKI control cohort, 142 of 688 (20.6%) patients had an ED visit, 316 of 688 (45.9%) were hospitalized, 36 of 688 (5.2%) were admitted to the ICU, 19 of 688 (2.8%) were mechanically ventilated/intubated, and 17 of 688 (2.5%) were dead. Comparatively, 147 of 688 (21.4%) patients with AKI had an ED visit (HR, 0.98; 0.78 to 1.24), 439 of 688 (63.8%) were hospitalized (HR, 1.60; 1.38 to 1.85), 129 of 688 (18.8%) were admitted to the ICU (HR, 3.86; 2.67 to 5.59), 141 of 688 (20.5%) were mechanically ventilated/intubated (HR, 8.22; 5.09 to 13.27), and 79 of 688 (11.5%) were deceased (HR, 4.81; 2.85 to 8.13).

#### At 180-Day Follow-Up

In the non-AKI control cohort, 204 of 688 (29.6%) patients had an ED visit, 330 of 688 (47.9%) were hospitalized, 51 of 688 (7.4%) were admitted to the ICU, 27 of 688 (3.9%) were mechanically ventilated/intubated, and 40 of 688 (5.8%) were deceased. Comparatively, 212 of 688 (30.8%) patients with AKI had an ED visit (HR, 1.10; 0.9 to 1.32), 455 of 688 (66.1%) were hospitalized (HR, 1.61; 1.39 to 1.85), 150 of 688 (21.8%) were admitted to the ICU (HR, 3.26; 2.37 to 4.47), 161 of 688 (23.4%) were mechanically ventilated/intubated (HR, 6.75; 4.49 to 10.15), and 129 of 688 (18.8%) were deceased (HR, 3.45; 2.42 to 4.93).

#### At 365-Day Follow-Up

**Figure 3:**
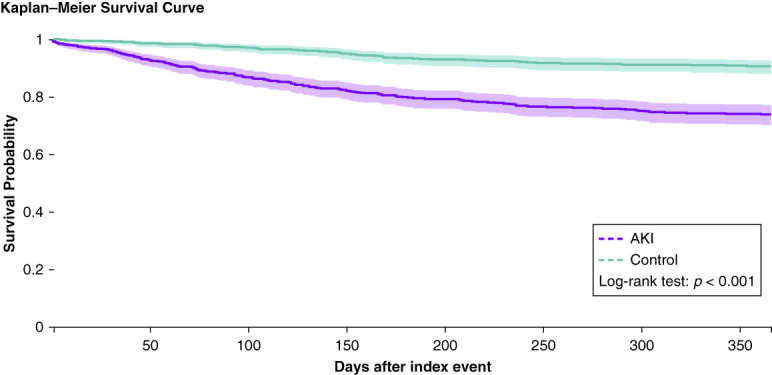
**Kaplan–Meier curve of all-cause mortality in patients with AKI (purple) compared with control patients (green) over 365 days.** Kaplan–Meier survival curves estimating the survival probability over 365 days. Survival probability at the end of time window was 73.86% for patients with AKI and 90.62% for controls.

In the non-AKI control cohort, 251 of 688 (36.5%) patients had an ED visit, 335 of 688 (48.7%) were hospitalized, 63 of 688 (9.2%) were admitted to the ICU, 31 of 688 (4.5%) were mechanically ventilated/intubated, and 56 of 688 (8.14%) were dead. Comparatively, 272 of 688 (39.5%) patients with AKI had an ED visit (HR, 1.20; 1.01 to 1.42), 466 of 688 (67.7%) were hospitalized (HR, 1.63; 1.42 to 1.88), 175 of 688 (25.4%) were admitted to the ICU (HR, 3.17; 2.37 to 4.23), 172 of 688 (25.0%) were mechanically ventilated/intubated (HR, 6.37; 4.35 to 9.34), and 163 of 688 (23.7%) were dead (HR, 3.19; 2.35 to 4.32). Kaplan–Meier curves are shown in Figure 3 and Supplemental Figures 2–5.

### Incidence and Prevalence of Dialysis

The findings are reported from 2013 to 2022. The incidence rates of dialysis in this population fluctuated, with the highest incidence observed in 2013 (6.10%) and the lowest in 2019 (2.76%). The prevalence of dialysis also exhibited variations over time, with the highest prevalence recorded in 2022 (12.67%) and the lowest in 2014 (4.70%).

**Figure 4:**
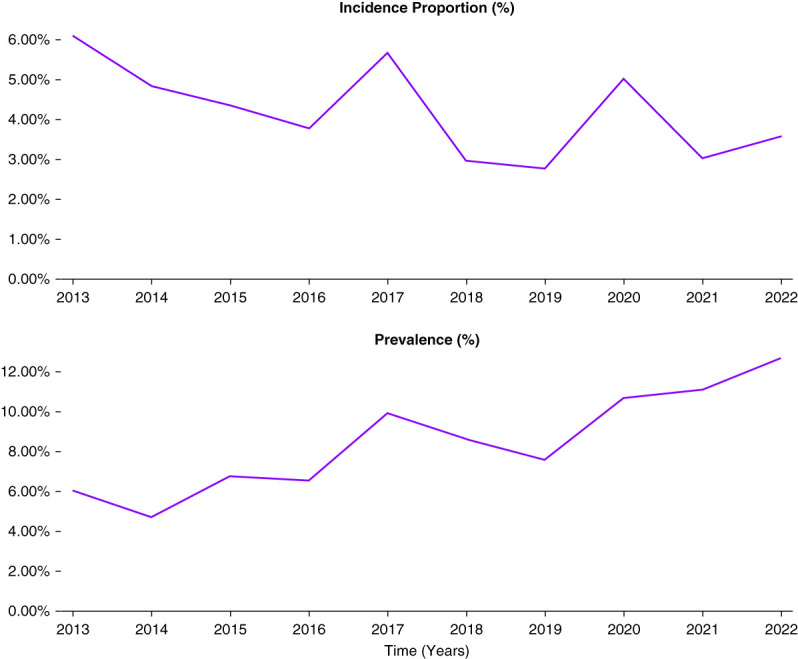
**Overall prevalence rate and incidence proportion of dialysis in patients with AKI over a 10-year span.** Incidence and prevalence values for HSCT-induced patients with AKI who went on to require dialysis services and procedure. Incidence proportion was as follows: 6.098% (2013), 4.831% (2014), 4.348% (2015), 3.759% (2016), 5.66% (2017), 2.959% (2018), 2.762% (2019), 5.013% (2020), 3.022% (2021), 3.571% (2022). Prevalence was as follows: 6.024% (2013), 4.695% (2014), 6.751% (2015), 6.522% (2016), 9.91% (2017), 8.611% (2018), 7.572% (2019), 10.67% (2020), 11.083% (2021), 12.668% (2022).

Among patients aged 0–4 years (mean serum creatinine 0.443), the incidence rate ranged from 7.75% in 2018 to 12.66% in 2022. In the 5–9 years age cohort (mean serum creatinine 0.486), the incidence varied from 6.94% in 2016 to 14.09% in 2013. Regarding prevalence rates, patients aged 0–4 years ranged from 8.0% in 2019 to 16.31% in 2017 and patients aged 5–9 years ranged from 7.41% in 2017 to 15.18% in 2022. Female patients generally experienced greater incidence rates, ranging from 6.25% in 2020 to 14.29% in 2013, compared with male patients, ranging from 5.07% in 2020 to 10.75% in 2013. Prevalence values were slightly more mixed, with female patients ranging from 8.22% in 2018 to 14.29% in 2013 and male patients ranging from 7.42% in 2019 to 14.88% in 2022. In 2022, the prevalence for male patients was 14.88% compared with 9.74% in female patients, Figure 4.

## Discussion

A compendium of literature exists on the pathophysiology and incidence of risk factors which precipitate AKI in pediatric patients after HSCT. However, data are limited on the comparative risks and clinical outcomes of the risk factors and medications or the need for dialysis. Our findings suggest the cumulative incidence of AKI diagnosis post-HSCT was 13.7%. We found the most common risk factors for AKI to be calcineurin inhibitors, hypertensive disease, and vancomycin. The risk factors which incurred the greatest mortality risk at 30 days were hypertensive disease (survival probability, 63.9%), calcineurin inhibitors (survival probability, 64.4%), and vancomycin (survival probability, 65.9%). HSCT patients with AKI were 1.7 times more likely to experience composite hospitalization and/or mortality 30 days from HSCT, with a mortality rate of 3.6% at 30 days and were at greater risk of all-cause hospitalization, ICU admission, mechanical ventilation/intubation, and mortality at 365 days.

The prevalence of dialysis has been increasing with time, but the annual incidence may in fact be decreasing. Incidence shows greater fluctuation on a year-to-year basis, but improved recognition of risk factors (*e.g*., nephrotoxic agents) may be contributing to an overall downtrend. Prevalence, in turn, may be bolstered by dialysis living longer because of early detection and intervention. It is imperative to develop prophylactic measures and screening; mortality post-HSCT in adult patients with AKI has been reported from 65% to 90%.^18,19^ Rates of hemodialysis in adults after HSCT has shown considerable variation. Kersting *et al.* retrospectively analyzed 363 patients who underwent allogeneic myeloablative stem cell transplantation and found that only 1.1% of patients went on to dialysis.^20^ Most recently, a cohort analysis conducted by Chapchap *et al.* found a 17.6% 1-year cumulative incidence of hemodialysis requirements, with progression to dialysis associated with male sex and age.^21^ Here, male patients showed a higher incidence of 14.88% compared with 9.74% in female patients. The cumulative incidence of postprocedure AKI was 13.7% in our study, consistent with data that analyzes AKI incidence 30 days after HSCT instead of 100 days after.^22^

Pediatric patients receiving HSCT, regardless of AKI diagnosis, had the greatest mortality risk after developing hypertension and/or hypertensive disease. Hypertensive sequelae were observed in 35.4% of patients who developed AKI, which is in concordance with the findings of Gülhan *et al.*, who reported 45% of patients had newly diagnosed hypertension early within the post-HSCT period.^23^ Early-onset hypertension has been reported in pediatric HSCT and is pronounced in younger patients; these findings are corroborated by our study.^24^ Despite the use of antihypertensive drugs, one third of these cases do not resolve at 2-year follow-up^25^ and may explain the poor reported survival probability. Hypertension is further complicated with a history of diabetes mellitus, which was observed in our data as a significant pretransplant risk factor, giving rise to endothelial dysfunction and metabolic abnormalities.^25^ Studies have demonstrated a significant benefit with the implementation of 24-hour ambulatory BP monitoring immediately after HSCT, as well as at regular intervals during the later periods post-HSCT.^26–28^

Many of the medications prescribed to patients after HSCT may induce hypertension. For instance, the incidence of calcineurin inhibitor–associated hypertension ranges from 65% to 100% in adult trials, which contextualizes the prevalence of calcineurin inhibitor use in our study and the poor survival probability.^29–31^ This trend has also been observed in patients administered cyclosporine (60%) and methotrexate (20%).^32^ It has been proposed that these drugs reduce nitric oxide availability and increase endothelin synthesis to subsequently increase kidney vascular tone.^33,34^ The diminishing integrity of the kidney vasculature may beget TMA, another complication of HSCT more common in patients on cyclosporine than tacrolimus.^35–37^ Moreover, calcineurin inhibitors have specifically been implicated in the activation of the renin-angiotensin system.^38^ In our study, patients with AKI experienced the second-lowest survival probability (64.4%) compared with non-AKI patients (83.5%) after being administered calcineurin inhibitors. Raina *et al.* recommended the replacement of immunosuppressants with less nephrotoxic agents, such as carboplatin. If calcineurin inhibitor use is necessary, tacrolimus is recommended.^39^ Gurbanov *et al.* recommended that immunosuppressants should be discontinued altogether in HSCT patients without GVHD.^26^

These recommendations may apply to the use of intravenous vancomycin, which is well-known for its nephrotoxic properties.^40^ In a retrospective study of stem cell transplant patients, 38% of those who developed AKI were administered vancomycin; two went on to require dialysis.^41^ Interestingly, AKI development was not associated with serum levels of vancomycin or dose/length of treatment. Several studies have reported that patients receiving vancomycin combined with piperacillin/tazobactam are at greater risk of developing AKI compared with any other antibiotic combination.^42–45^ Clemmons *et al.*^43^ reported that the incidence of AKI was 68% in patients administered vancomycin with piperacillin/tazobactam because of tubular ischemia from oxidative stress. This phenomenon was observed in both the engraftment and pre-engraftment periods.^46^ In a sustained prospective quality improvement program to reduce the impact of drug-induced nephrotoxicity in pediatric patients, Goldstein *et al.* implemented an EHR screening and decision support process with daily serum creatinine monitoring over 43 months. The exposure rate of nephrotoxic agents decreased 38% and incidence of AKI decreased 64% from baseline, avoiding 633 exposures and 398 AKI episodes.^40^ More specifically in stem cell transplant patients, Hambrick *et al.* implemented an antimicrobial stewardship program to reduce prolonged vancomycin use (> 72 hours) and thereby reduced the rate of AKI in pediatric patients by 37%.^47^

## Limitations

This cohort study is observational and retrospective in nature, which allows for understanding of trends and associations but not causation. Moreover, data may be susceptible to inaccurate or incomplete coding and depends on the quality of documentation in the EHR. This may be exacerbated by variations in AKI or hypertension definition/diagnosis. However, TriNetX employs a rigorous data cleaning process which incorporates data updates and presence of required fields.^48^

TriNetX employs R survival package to generate HR, which may be susceptible to potential clustering of AKI cases within specific health care organizations from which data are aggregated. The risk analysis feature in TriNetX is also limited to a maximum of ten variables, making a more comprehensive analysis unfeasible. Finally, despite propensity matching for demographic and clinical variables, inappropriate confounding variables may be present and influence the results of the severity analysis.

In conclusion, our findings suggest subsequent hypertensive disease, calcineurin inhibitor use, and vancomycin use were associated with the greatest cumulative incidence and posed the greatest mortality risk. Practices such as 24-hour BP monitoring and discontinuation of the potential nephrotoxic medications have shown prior success. Further large-scale research is needed to understand mortality risk associated with different stages of AKI and effective interventions.

## Supplementary Material

## Data Availability

All data are included in the manuscript and/or supporting information. Data cannot be shared. The platform employed in this study aggregates deidentified patient data which are cloud-based and, therefore, cannot be downloaded and/or exported. The platform also provides data in real time, meaning the patient records shown are regularly updated and changed to reflect the most current diagnoses, procedures, medications, and so on. To mediate this, the authors provide specific time frames and network description to bring light to which data set was used.

## Supplemental Material

Supplemental Methods. Database description and cohort criteria.

Supplemental Table 1. Covariates used for propensity score matching.

Supplemental Table 2. Hazard ratios of transplant-related risk factors.

Supplemental Table 3. Hazard ratios of clinical outcomes at 90-day, 180-day, and 365-day intervals.

Supplemental Table 4. Current procedural terminology (CPT) codes used to quantify severity of clinical outcomes.

Supplemental Figure 1. Propensity score density function.

Supplemental Figure 2. Kaplan–Meier curves of AKI and control cohorts after all-cause emergency department visit.

Supplemental Figure 3. Kaplan–Meier curves of AKI and control cohorts after all-cause hospitalization.

Supplemental Figure 4. Kaplan–Meier curves of AKI and control cohorts after all-cause intensive care unit admission.

Supplemental Figure 5. Kaplan–Meier curves of AKI and control cohorts after all-cause mechanical ventilation/intubation.
