# Supplementary material for: Epidemiology, Risk Factors, and Clinical Outcomes of AKI in Pediatric Hematopoietic Stem Cell Transplant Patients
Source: Kidney360. 2024 Mar 7;5(6):802–11. doi: 10.34067/KID.0000000000000410 (PMC11219119; doi:10.34067/KID.0000000000000410)
Supplement: Supplementary file 1 [file kidney360-5-802-s001.pdf]

Supplementary Material

Supplemental Methods. Database description and cohort criteria

Supplemental Table 1. Covariates used for propensity-score matching

Supplemental Table 2. Hazard ratios of transplant-related risk factors

Supplemental Table 3. Hazard ratios of clinical outcomes at 90-, 180-, and 365-day intervals

Supplemental Table 4. Current Procedural Terminology (CPT) codes utilized to quantify severity of clinical outcomes

Supplemental Figure 1. Propensity Score Density Function

Supplemental Figure 2. Kaplan-Meier curves of AKI and control cohorts after all-cause emergency department visit

Supplemental Figure 3. Kaplan-Meier curves of AKI and control cohorts after all-cause hospitalization

Supplemental Figure 4. Kaplan-Meier curves of AKI and control cohorts after all-cause intensive care unit admission

Supplemental Figure 5. Kaplan-Meier curves of AKI and control cohorts after all-cause mechanical ventilation/intubation

Supplemental Methods

Database Description of TriNetX Analytics Platform:
The data used in this study was collected from the TriNetX Global Collaborative Network which provides access to electronic health records, including encounter diagnoses, procedures, medications, laboratory values, and genomic information, from approximately 137 million patients from 108 healthcare organizations. All analyses were run on June 23, 2023. TriNetX, LLC is compliant with the Health Insurance Portability and Accountability Act (HIPAA), the US federal law which protects the privacy and security of healthcare data, and any additional data privacy regulations applicable to the contributing healthcare organizations. Studies utilizing TriNetX data as described here have been exempt from review by the Case Western Reserve University/MetroHealth System, Cleveland Ohio Institutional Review Board; the patient data described is aggregated, deidentified data, and not Human Subject Research.

TriNetX Analytics Platform de-identifies and aggregates electronic health record (EHR) data from 108 contributing healthcare organizations, most of which are large academic medical institutions with both inpatient and outpatient facilities at multiple locations, accounting for more than 20% of the United States population and across 14 other countries. TriNetX Analytics offers a secure and cloud-based platform for accessing electronic health record data of patients. This includes data from hospitals, primary care facilities, and specialty treatment providers across various geographic locations. The database encompasses a diverse range of age groups, racial and ethnic backgrounds, income levels, and insurance types. These insurance types include commercial insurances, governmental insurance, Center of Medicare and Medicaid Service programs, Veterans Affairs insurance, self-pay or uninsured cases, worker compensation insurance, and others. The database solely contains EHR data and does not link to or provide information on claims data. Any data that may resemble claims information originates from the EHR system itself, which also serves the purpose of generating claims.

Statistical Analysis:

Measures of association were used to determine risk difference with associated 95% confidence intervals (95% CI). Competing risks analysis was used to illustrate likelihood of experiencing mutually exclusive outcomes by calculating Cumulative Incidence at End of Time Window and Aalen-Johansen curve using the R Survival library, version 3.2-3. The competing risks analysis evaluated risk factors independently using an Aalen-Johansen estimator which reduces to the Kaplan-Meier estimator to determine cumulative incidence. Cox proportional hazards regression analysis was used to compare time-to-event rates in matched cohorts. Kaplan-Meier analysis was used to estimate the survival probability of given clinical outcomes. Hazard ratio (HR) and associated 95% CI were calculated using R's Survival package, version 3.2-3. The incidence proportion of dialysis requirement (CPT codes: 1012740, 1029674 90945, 90947; Systemized Nomenclature of Medicine code: 108241001; ICD-10 code: Z99.2) was defined as the rate of new cases of interest divided by the number of patients who do not have the event of interest during the given time window annually. Prevalence rate was defined as the rate of all cases recorded within the lookback period. Significance tests were 2-sided, paired, and statistical significance was set at $p < .05$.

Supplemental Table 1. Covariates used for propensity score matching

Covariate	Demographic Code	Data Type
Age	-	Continuous
Female	-	Present/Absent
Male	-	Present/Absent
Asian	2028-9	Present/Absent
Black or African American	2054-5	Present/Absent
White	2106-3	Present/Absent
Other Race	2131-1	Present/Absent
Hypertension	I10	Present/Absent
Diabetes mellitus	E08-E13	Present/Absent
Chronic kidney disease	N18	Present/Absent
Proteinuria	R80	Present/Absent
Neoplasia	C00-D49	Present/Absent

All analyses were performed June 23rd, 2023.

Supplemental Table 2. Hazard ratios of transplant-related risk factors

	AKI % (n)	Control % (n)	Hazard Ratio (95% CI)
**Transplant-Related Risk Factors**			
Hypertension/ Hypertensive disease	35.43% (214)	8.44% (51)	**4.86 (3.58-6.60)**
Hepatic veno-occlusive disease	4.31% (26)	1.66% (10)	**4.37 (1.80-10.61)**
Thrombotic microangiopathy	4.97% (30)	1.66% (10)	**5.07 (2.11-12.19)**
Graft-versus-host disease	20.36% (123)	3.15% (19)	**7.05 (4.35-11.44)**
Sepsis	13.08% (79)	2.98% (18)	**4.57 (2.74-7.63)**
Immunodeficiency due to drugs and external causes	9.44% (57)	4.31% (26)	**2.22 (1.40-3.54)**
Vancomycin	33.44% (202)	25.17% (152)	**1.41 (1.14-1.74)**
Foscarnet	6.29% (38)	2.15% (13)	**2.96 (1.58-5.56)**
Etoposide	4.64% (28)	2.15% (13)	**2.16 (1.12-4.17)**
Aminoglycosides	11.42% (69)	4.80% (29)	**2.44 (1.58-3.76)**
Calcineurin inhibitors	34.93% (211)	15.89% (96)	**2.56 (2.01-3.26)**
Intravenous Immunoglobulin G	24.34% (147)	12.42% (75)	**2.08 (1.57-2.74)**
Methotrexate	8.11% (49)	8.94% (54)	**0.89 (0.61-1.31)**

Hazard ratios of transplant-related risk factors calculated from Cox proportional hazards model. Encounter diagnosis of graft-versus-host disease was shown to incur the greatest hazard rate.

Supplemental Table 3. Hazard ratios of clinical outcomes at 90-, 180-, and 365-day intervals

Clinical Outcomes	Hazard Ratio (95% CI)
**90-Day**	
ED Visit	0.98 (0.78-1.24)
Hospitalization	1.60 (1.38-1.85)
ICU Admission	3.86 (2.67-5.59)
Mechanical Ventilation/Intubation	8.22 (5.09-13.27)
Mortality	4.81 (2.85-8.13)
**180-Day**	
ED Visit	1.10 (0.9-1.32)
Hospitalization	1.61 (1.39-1.85)
ICU Admission	3.26 (2.37-4.47)
Mechanical Ventilation/Intubation	6.75 (4.49-10.15)
Mortality	3.45 (2.42-4.93)
**365-Day**	
ED Visit	1.20 (1.01-1.42)
Hospitalization	1.63 (1.42-1.88)
ICU Admission	3.17 (2.37-4.23)
Mechanical Ventilation/Intubation	6.37 (4.35-9.34)
Mortality	3.19 (2.35-4.32)

ED: emergency department; ICU: intensive care unit. Hazard ratios were calculated from Cox proportional hazards model.

Supplemental Table 4. Current Procedural Terminology (CPT) codes utilized to quantify severity of clinical outcomes

Outcomes	Current Procedural Terminology Code
Emergency Department Visit	1013711: Emergency Department Services<br>1013712: New or Established Patient<br>1013723: Other Emergency Services
Hospitalization	1013659: Hospital Inpatient Services<br>1013660: Initial Hospital Care<br>1013699: Inpatient Consultations<br>99221: Initial hospital care, per day, for the evaluation and management of a patient, which requires these 3 key components: A detailed or comprehensive history; A detailed or comprehensive examination; and Medical decision making that is straightforward or of low complexity. Counseling and/or coordination of care with other physicians, other qualified health care professionals, or agencies are provided consistent with the nature of the problem(s) and the patient's and/or family's needs. Usually, the problem(s) requiring admission are of low severity. Typically, 30 minutes are spent at the bedside and on the patient's hospital floor or unit.<br>99222: Initial hospital care, per day, for the evaluation and management of a patient, which requires these 3 key components: A comprehensive history; A comprehensive examination; and Medical decision making of moderate complexity. Counseling and/or coordination of care with other physicians, other qualified health care professionals, or agencies are provided consistent with the nature of the problem(s) and the patient's and/or family's needs. Usually, the problem(s) requiring admission are of moderate severity. Typically, 50 minutes are spent at the bedside and on the patient's hospital floor or unit.<br>99223: Initial hospital care, per day, for the evaluation and management of a patient, which requires these 3 key components: A comprehensive history; A comprehensive examination; and Medical decision making of high complexity. Counseling and/or coordination of care with other physicians, other qualified health care professionals, or agencies are provided consistent with the nature of the problem(s) and the patient's and/or family's needs. Usually, the problem(s) requiring admission are of high severity. Typically, 70 minutes are spent at the bedside and on the patient's hospital floor or unit.<br>99224: Subsequent observation care, per day, for the evaluation and management of a patient, which requires at least 2 of these 3 key components: Problem focused interval history; Problem focused examination; Medical decision making that is straightforward or of low complexity. Counseling and/or coordination of care with other physicians, other qualified health care professionals, or agencies are provided consistent with the nature of the problem(s) and the patient's and/or family's needs. Usually, the patient is stable, recovering, or improving. Typically, 15 minutes are spent at the bedside and on the patient's hospital floor or unit.

	99225: Subsequent observation care, per day, for the evaluation and management of a patient, which requires at least 2 of these 3 key components: An expanded problem focused interval history; An expanded problem focused examination; Medical decision making of moderate complexity. Counseling and/or coordination of care with other physicians, other qualified health care professionals, or agencies are provided consistent with the nature of the problem(s) and the patient's and/or family's needs. Usually, the patient is responding inadequately to therapy or has developed a minor complication. Typically, 25 minutes are spent at the bedside and on the patient's hospital floor or unit.<br>99226: Subsequent observation care, per day, for the evaluation and management of a patient, which requires at least 2 of these 3 key components: A detailed interval history; A detailed examination; Medical decision making of high complexity. Counseling and/or coordination of care with other physicians, other qualified health care professionals, or agencies are provided consistent with the nature of the problem(s) and the patient's and/or family's needs. Usually, the patient is unstable or has developed a significant complication or a significant new problem. Typically, 35 minutes are spent at the bedside and on the patient's hospital floor or unit.<br>99231: Subsequent hospital care, per day, for the evaluation and management of a patient, which requires at least 2 of these 3 key components: A problem focused interval history; A problem focused examination; Medical decision making that is straightforward or of low complexity. Counseling and/or coordination of care with other physicians, other qualified health care professionals, or agencies are provided consistent with the nature of the problem(s) and the patient's and/or family's needs. Usually, the patient is stable, recovering or improving. Typically, 15 minutes are spent at the bedside and on the patient's hospital floor or unit.<br>99232: Subsequent hospital care, per day, for the evaluation and management of a patient, which requires at least 2 of these 3 key components: An expanded problem focused interval history; An expanded problem focused examination; Medical decision making of moderate complexity. Counseling and/or coordination of care with other physicians, other qualified health care professionals, or agencies are provided consistent with the nature of the problem(s) and the patient's and/or family's needs. Usually, the patient is responding inadequately to therapy or has developed a minor complication. Typically, 25 minutes are spent at the bedside and on the patient's hospital floor or unit.<br>99233: Subsequent hospital care, per day, for the evaluation and management of a patient, which requires at least 2 of these 3 key components: A detailed interval history; A detailed examination; Medical decision making of high complexity. Counseling and/or coordination of care with other physicians, other qualified health care professionals, or agencies are provided consistent with the nature of the problem(s) and the patient's and/or family's needs. Usually, the patient is unstable or has developed a significant complication or a significant new problem.

	Typically, 35 minutes are spent at the bedside and on the patient's hospital floor or unit.
Intensive Care Unit Admission	1013729: Critical Care Services<br>1014309: Critical care, evaluation and management of the critically ill or critically injured patient
Mechanical Ventilation/Intubation	94002: Ventilation assist and management, initiation of pressure or volume preset ventilators for assisted or controlled breathing; hospital inpatient/observation, initial day<br>94003: Ventilation assist and management, initiation of pressure or volume preset ventilators for assisted or controlled breathing; hospital inpatient/observation, each subsequent day<br>31500: Intubation, endotracheal, emergency procedure<br>1015098: Ventilator Management<br>1022227: Extracorporeal membrane oxygenation (ECMO)/extracorporeal life support (ECLS) provided by physician<br><br>ICD-10-PCS Codes<br>5A1945Z: Respiratory Ventilation, 24 Consecutive Hours<br>5A09557: Respiratory Ventilation, Greater than 96 Consecutive Hours<br>5A09357: Respiratory Ventilation, Less than 24 Consecutive Hours<br>0BH13EZ: Insertion of Endotracheal Airway into Trachea, Percutaneous Approach<br>0BH17EZ: Insertion of Endotracheal Airway into Trachea, Via Natural or Artificial Opening<br>0BH18EZ: Insertion of Endotracheal Airway into Trachea, Via Natural or Artificial Opening Endoscopic

Supplemental Figure 1. Propensity Score Density Function

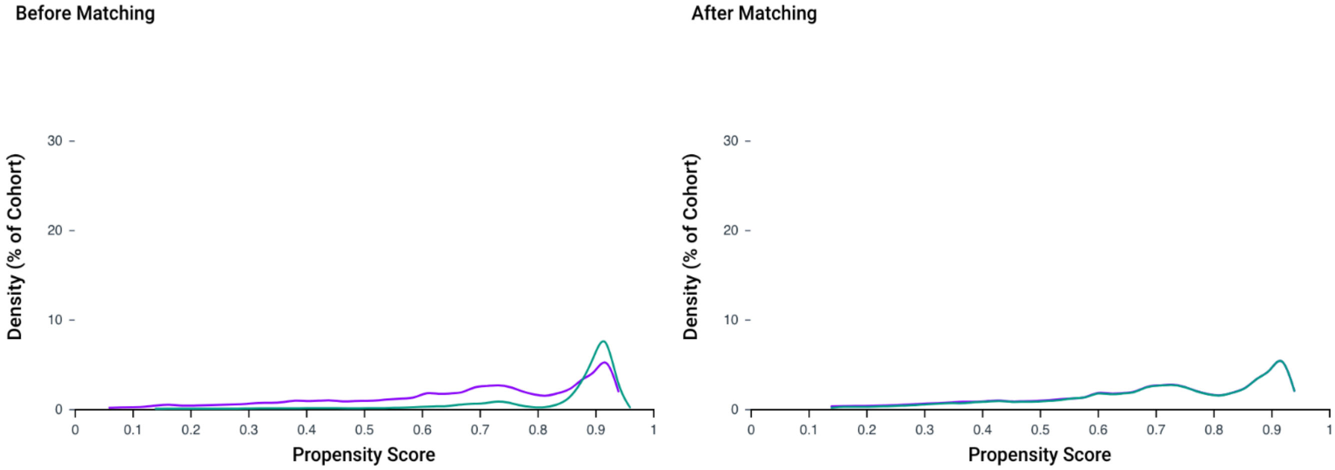

1:1 propensity score matching was conducted using a greedy nearest-neighbor matching algorithm with an arbitrary tolerance level set at 0.1. Purple indicates AKI patients and green indicates non-AKI patients.

Supplemental Figure 2. Kaplan-Meier curves of AKI and control cohorts after all-cause emergency department visit

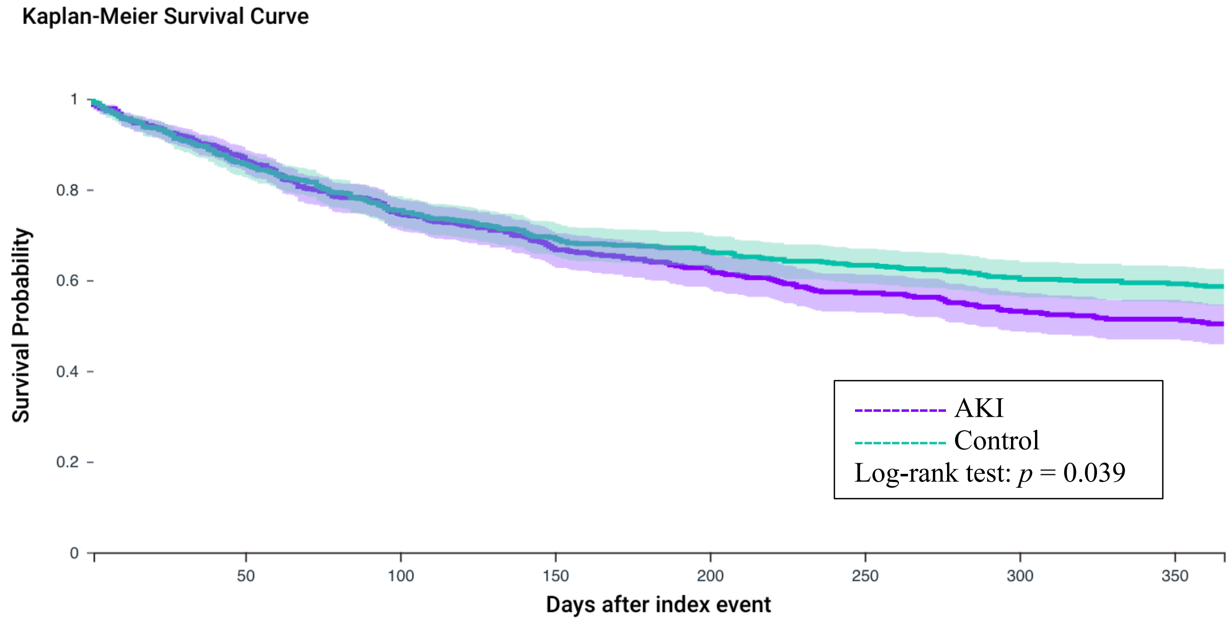

Kaplan-Meier survival curves estimating the survival probability after emergency department visit over 365 days. Survival probability at the end of time window was 50.34% for AKI patients and 58.59% for control patients.

Supplemental Figure 3. Kaplan-Meier curves of AKI and control cohorts after all-cause hospitalization

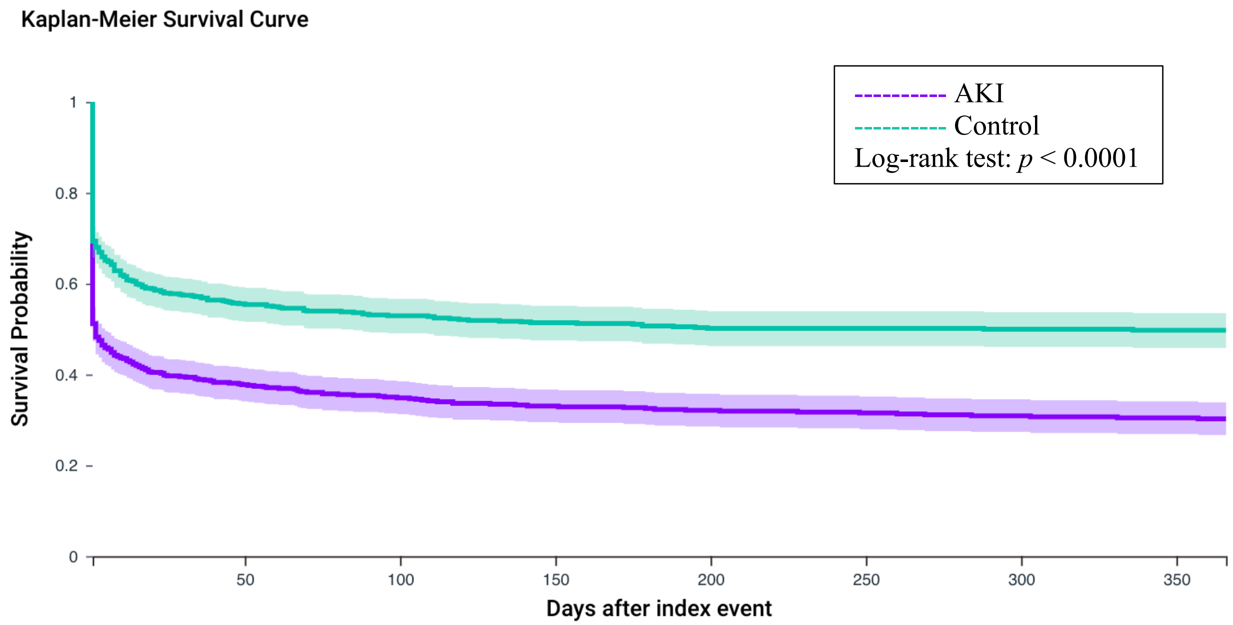

Kaplan-Meier survival curves estimating the survival probability after inpatient hospitalization over 365 days. Survival probability at the end of time window was 30.22% for AKI patients and 49.72% for control patients.

Supplemental Figure 4. Kaplan-Meier curves of AKI and control cohorts after all-cause intensive care unit admission

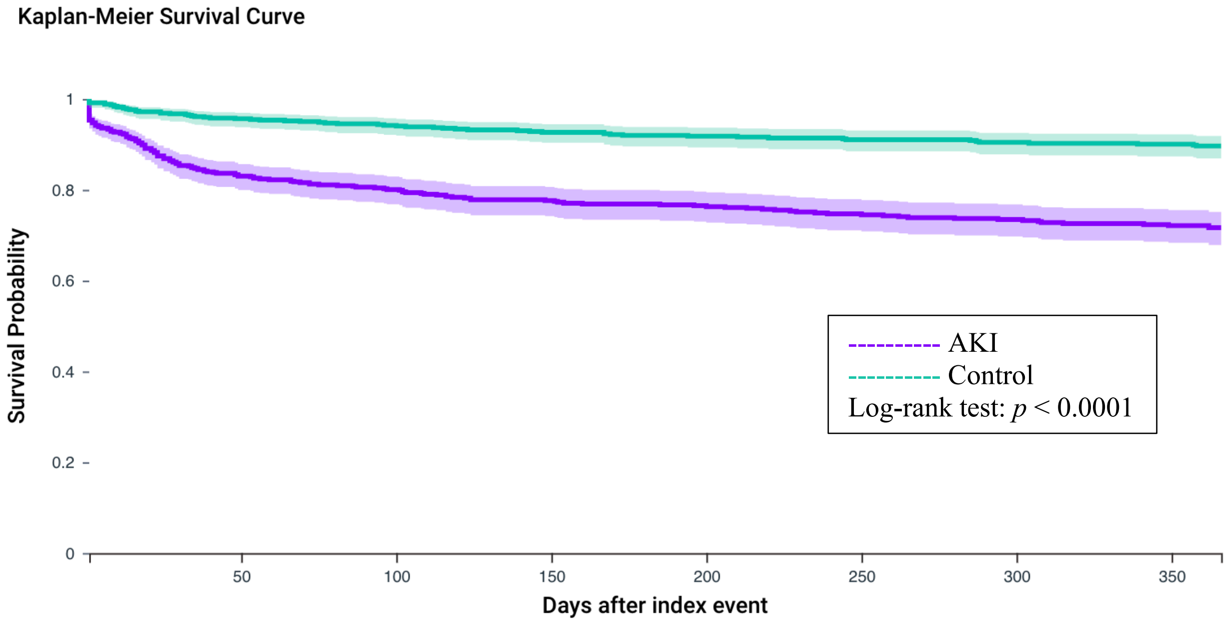

Kaplan-Meier survival curves estimating the survival probability after intensive care unit admission over 365 days. Survival probability at the end of time window was 71.64% for AKI patients and 89.63% for control patients.

Supplemental Figure 5. Kaplan-Meier curves of AKI and control cohorts after all-cause mechanical ventilation/intubation

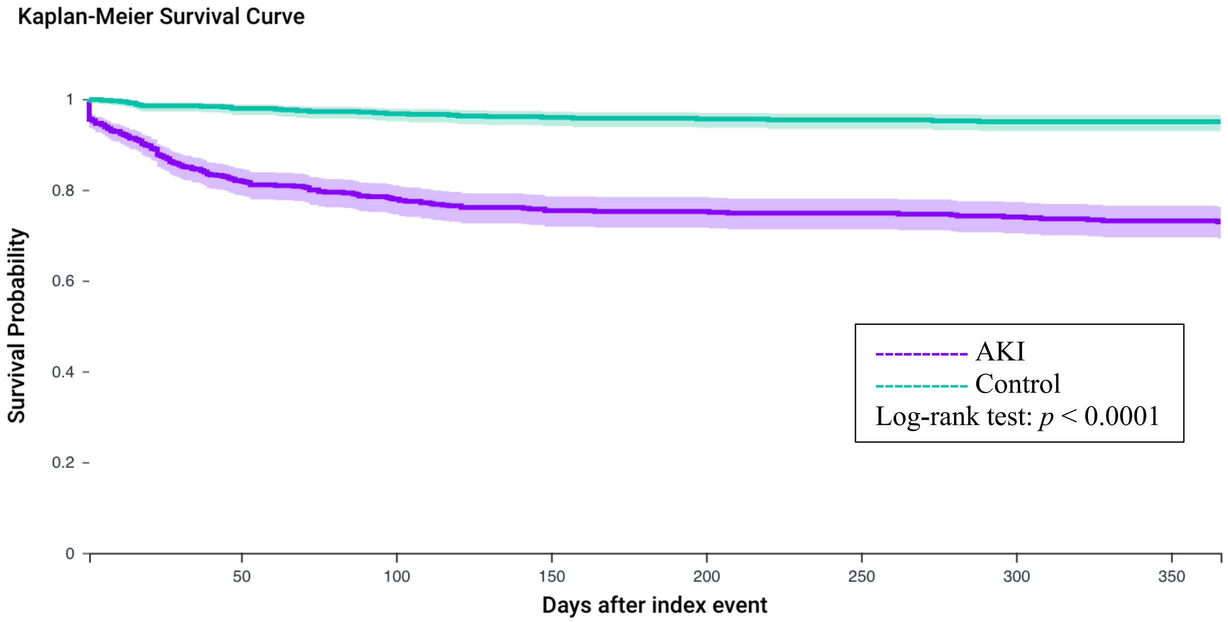

Kaplan-Meier survival curves estimating the survival probability after intensive care unit admission over 365 days. Survival probability at the end of time window was 72.92% for AKI patients and 94.98% for control patients.
